# Exploration of a hypoxia-immune-related microenvironment gene signature and prediction model for hepatitis C-induced early-stage fibrosis

**DOI:** 10.1186/s12967-024-04912-6

**Published:** 2024-01-29

**Authors:** Chuwen Chen, Haozheng Cai, Junyi Shen, Xiaoyun Zhang, Wei Peng, Chuan Li, Haopeng Lv, Tianfu Wen

**Affiliations:** 1 Division of Liver Surgery, Department of General Surgery, West China Hospital, Si Chuan University, Chengdu, 610041 China; 2 Department of General Surgery, ChengDu Shi Xinjin Qu Renmin Yiyuan: People’s Hospital of Xinjin District, Chengdu, China

**Keywords:** HCV, Liver fibrosis, Hypoxia, Immune, Prognosis

## Abstract

**Background:**

Liver fibrosis contributes to significant morbidity and mortality in Western nations, primarily attributed to chronic hepatitis C virus (HCV) infection. Hypoxia and immune status have been reported to be significantly correlated with the progression of liver fibrosis. The current research aimed to investigate the gene signature related to the hypoxia-immune-related microenvironment and identify potential targets for liver fibrosis.

**Method:**

Sequencing data obtained from GEO were employed to assess the hypoxia and immune status of the discovery set utilizing UMAP and ESTIMATE methods. The prognostic genes were screened utilizing the LASSO model. The infiltration level of 22 types of immune cells was quantified utilizing CIBERSORT, and a prognosis-predictive model was established based on the selected genes. The model was also verified using qRT-PCR with surgical resection samples and liver failure samples RNA-sequencing data.

**Results:**

Elevated hypoxia and immune status were linked to an unfavorable prognosis in HCV-induced early-stage liver fibrosis. Increased plasma and resting NK cell infiltration were identified as a risk factor for liver fibrosis progression. Additionally, *CYP1A2, CBS, GSTZ1, FOXA1, WDR72* and *UHMK1* were determined as hypoxia-immune-related protective genes. The combined model effectively predicted patient prognosis. Furthermore, the preliminary validation of clinical samples supported most of the conclusions drawn from this study.

**Conclusion:**

The prognosis-predictive model developed using six hypoxia-immune-related genes effectively predicts the prognosis and progression of liver fibrosis. The current study opens new avenues for the future prediction and treatment of liver fibrosis.

## Introduction

Liver fibrosis contributes to significant morbidity and mortality in Western countries, primarily attributed to chronic viral hepatitis C infection. Hepatitis C virus (HCV) impacts around 200 million individuals globally, significantly burdening healthcare systems [1]. The severity of liver fibrosis impacts liver function and increases the risk of developing hepatocellular carcinoma (HCC) [2]. Moreover, liver fibrosis leads to various complications, such as chronic portal hypertension, bleeding, ascites, encephalopathy and jaundice, severely impairing the quality of life of patients [3]. While liver transplantation remains the most effective treatment for liver fibrosis, its limited availability and high cost hinder its widespread adoption among patients with liver fibrosis. Despite advances in the treatment of HCV in recent year, the treatment of HCV-induced liver cirrhosis remains challenging. There are currently no approved medications for HCV-induced liver fibrosis. Therefore, identifying new drug targets for preventing and treating HCV-induced liver fibrosis is a vital need.

Oxygen availability is crucial for hepatocyte survival, and its deficiency has been documented as a major risk factor for liver diseases [4]. Liver fibrosis is distinguished by the overaccumulation of the extracellular matrix (ECM) [5]. However, the deposition of ECM in the liver increases vascular resistance, impeding the supply of oxygenated blood to the liver, including the portal vein, thereby aggravating liver hypoxia [6]. Moreover, hypoxia has been significantly correlated with liver fibrosis. Therefore, exploring the role of hypoxia in liver fibrosis can enhance understanding of its progression of fibrosis and can aid in identifying novel therapeutic targets. The objective of this research is to investigate the impact of hypoxia and immune status on the prognosis of individuals with early-stage liver fibrosis caused by HCV infection. Additionally, the study seeks to identify a panel of hypoxia-immune-related genes that may offer potential diagnostic and therapeutic targets for HCV-induced fibrosis.

## Materials and method

**Fig. 1:**
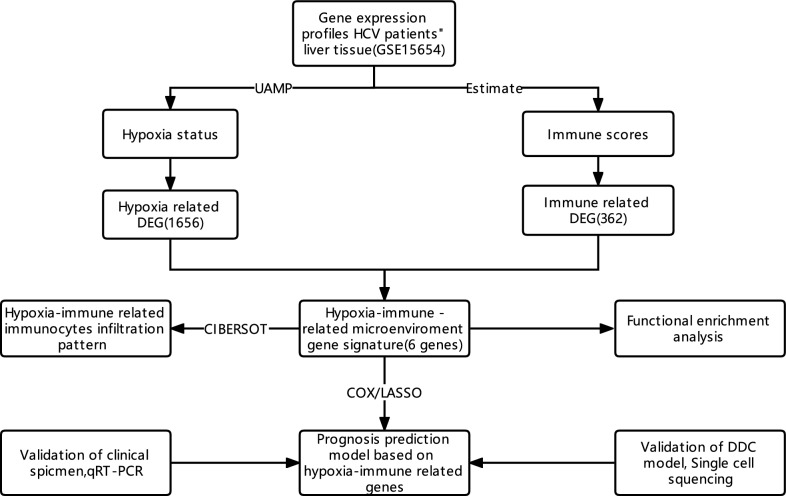
The ideas and analysis process of our research

The study design is presented in Fig. 1.

### Patient cohort and data preparation

The discovery cohort of participants in the research comprised 216 individuals with hepatitis C-related early-stage (Child–Pugh class A) cirrhosis obtained from the Gene Expression Omnibus (GEO) database (GSE15654) (https://www.ncbi.nlm.nih.gov/geo/query/acc.cgi?acc=gse15654). The sequencing samples comprised formalin-fixed needle biopsy specimens obtained from the livers of affected individuals. The microarray data from GSE15654 were generated using the GPL8432 platform (Illumina HumanRef-8 WG-DASL v3.0), resulting in an original matrix of 24,526 genes. Duplicated genes were removed, and the max value was retained, resulting in 18,392 genes. The matrix was then normalized through log2 transformation. The features of the affected individuals in the discovery cohort are presented in Table 1.

**Table 1:** Basic information of HCV patients in discovery cohort.

Characteristics	GSE15654(n = 216)
Survival	150 (69.44%)
Survival time (days)	3373.13 ± 1525.10
Bilirubin ≥ 1.0 mg/dl	108 (50.00%)
HCC	65 (30.09)

*HCC* Hepatic carcinoma, *HCV*: hepatitis C virus

### Detection of hypoxia status and hypoxia-related differentially expressed genes (DEGs)

A collection of 200 hallmark genes related to hypoxia was acquired from the Molecular Signatures Database (MSigDB version 6.0). The non-linear dimensionality reduction algorithm, Uniform Manifold Approximation and Projection (UMAP), was employed to categorize the affected individuals into a high-hypoxia group (hypoxia^high^) and low-hypoxia group (hypoxia^low^) based on the expression of hypoxia genes. UMAP analysis was carried out with the aid of the package ‘Seurat’. The Kaplan-–Meier method was utilized to generate a survival plot. The limma algorithm was implemented to detect DEGs. Genes with a false discovery rate (FDR) adjusted *P*-value < 0.05 and an absolute value of log2 (fold change) > 0.5 were classified as hypoxia-related DEGs.

### Detection of immune status and immune-related DEGs

The Estimation of Stromal and Immune cells in MAlignant Tumours using the Expression data (ESTIMATE) algorithm was used to identify the immune status of patients through the package ‘estimate’. Individuals were divided into two groups using Maximally Selected Rank Statistics, according to their immune scores (immune^high^ and immune^low^). The Kaplan–Meier method was employed to generate a survival plot. DEGs between the two groups were detected utilizing the limma algorithm. Genes with an FDR-adjusted *P*-value < 0.05 and an absolute value of log2 (fold change) > 0.5 were classified as immune-related DEGs. Maximally selected rank statistics and survival plots were performed using the R packages ‘survival’ and ‘survminer’.

### Identification of hypoxia-immune-related prognostic DEGs

Based on the immune and hypoxia status of affected individuals, three groups were established (hypoxia^high^immune^high^, hypoxia^low^immune^low^, and mix). DEGs between the hypoxia^high^immune^high^ and hypoxia^low^immune^low^ groups were detected using the limma algorithm. Genes with an FDR-adjusted *P*-value < 0.05 and an absolute value of log2 (fold change) > 0.5 were categorized as hypoxia-immune-related prognostic DEGs. Subsequently, the overlap between immune-related DEGs and hypoxia-related DEGs was retained, and univariate Cox analysis was carried out to choose DEGs with a *P* < 0.01 for further analysis.

### Prognosis prediction model of hcv-related early-stage fibrosis as per the hypoxia-immune-related DEGs

Following the univariate Cox analysis, the least absolute shrinkage and selection operator (LASSO), a form of linear regression with shrinkage, was employed to screen genes. The corresponding coefficients were quantified utilizing multivariate Cox regression. Using the R package ‘glmnet’, both LASSO and Cox regression analyses were carried out. The risk scores were calculated using the formula: Score = 1nCoefi ∗ the expression of the relative gene. Maximally selected rank statistics were employed to categorize the affected individuals into two groups (high-risk group and low-risk group) as per their risk scores. Survival plots were created to assess the predictive utility of our prognosis prediction model.

### Functional and pathway enrichment analysis

The functions and signaling pathways of hypoxia-related DEGs, immune-related DEGs, and hypoxia-immune-related DEGs were identified by means of Gene Ontology (GO) functional annotation analysis and Kyoto Encyclopedia of Genes and Genomes (KEGG) analysis. The GO and KEGG analyses were carried out using https://david.ncifcrf.gov/home.jsp.

### Identification of immune cells infiltration in HCV-related early-stage fibrosis

CIBERSORT analysis was performed to identify the infiltration of 22 immune cells in patients with HCV-related early-stage cirrhosis. Pearson analysis was conducted to investigate the association between the infiltration of immune cells and the expression of hypoxia-immune DEGs. Additionally, the variations in immune cell infiltration between the high-risk and low-risk prognosis groups were assessed. Cells with a *P* < 0.01 were retained for display in this research. CIBERSORT was carried out by employing the R package ‘glmnet’, ‘parallel’,’preprocessCore’, ‘tidyr’, ‘ggplot2’, and ‘ggpubr’.

### Verification of clinical samples

The surgical liver specimens were obtained from twelve individuals at the time of liver transplantation, including six individuals with liver failure and six liver tissue samples from donors after cardiac death. Seven cirrhotic samples were obtained from patients with laparoscopic splenectomy and esophagogastric devascularization surgery. The approval of the research were provided by the West China Hospital, Sichuan University. Quantitative reverse transcriptase-PCR (qRT-PCR) was conducted to detect the expression of hypoxia-immune DEGs. TRIzol Reagent was utilized to extract the total RNA from the liver tissues. PCR was conducted utilizing the Thermo Scientific PikoReal PCR cycler. The mean cycle threshold (CT) data was calculated from triplicate PCRs. The equation  2^−△CT^ was employed to determine the relative gene expression.

### RNA-seq analysis of liver failure samples

Thirty-six liver failure specimens were obtained from patients undergoing liver transplantation. Hepatitis B virus and hepatitis C virus were the mainly etiology. Total RNA was extracted from the liver specimens with the use of an RNA Isolation Kit (Foregene, Chengdu, China). Construction of RNA-sequencing (RNA-seq) libraries and sequencing with the Illumina NovaSeq™ X Plus platform were performed by Anoroad (Beijing, China). Relative gene expression was evaluated on the basis of transcripts per million (TPM).

### Statistical analysis

All analyses were carried out using R version 4.0.2 and Graphad Prim 9.0. An unpaired t-test was utilized to compare continuous data, and data not conforming to a normal distribution were assessed using Mann–Whitney U test.

## Results

### High hypoxia status indicated poor prognosis in HCV-induced early-stage fibrosis.

**Fig. 2:**
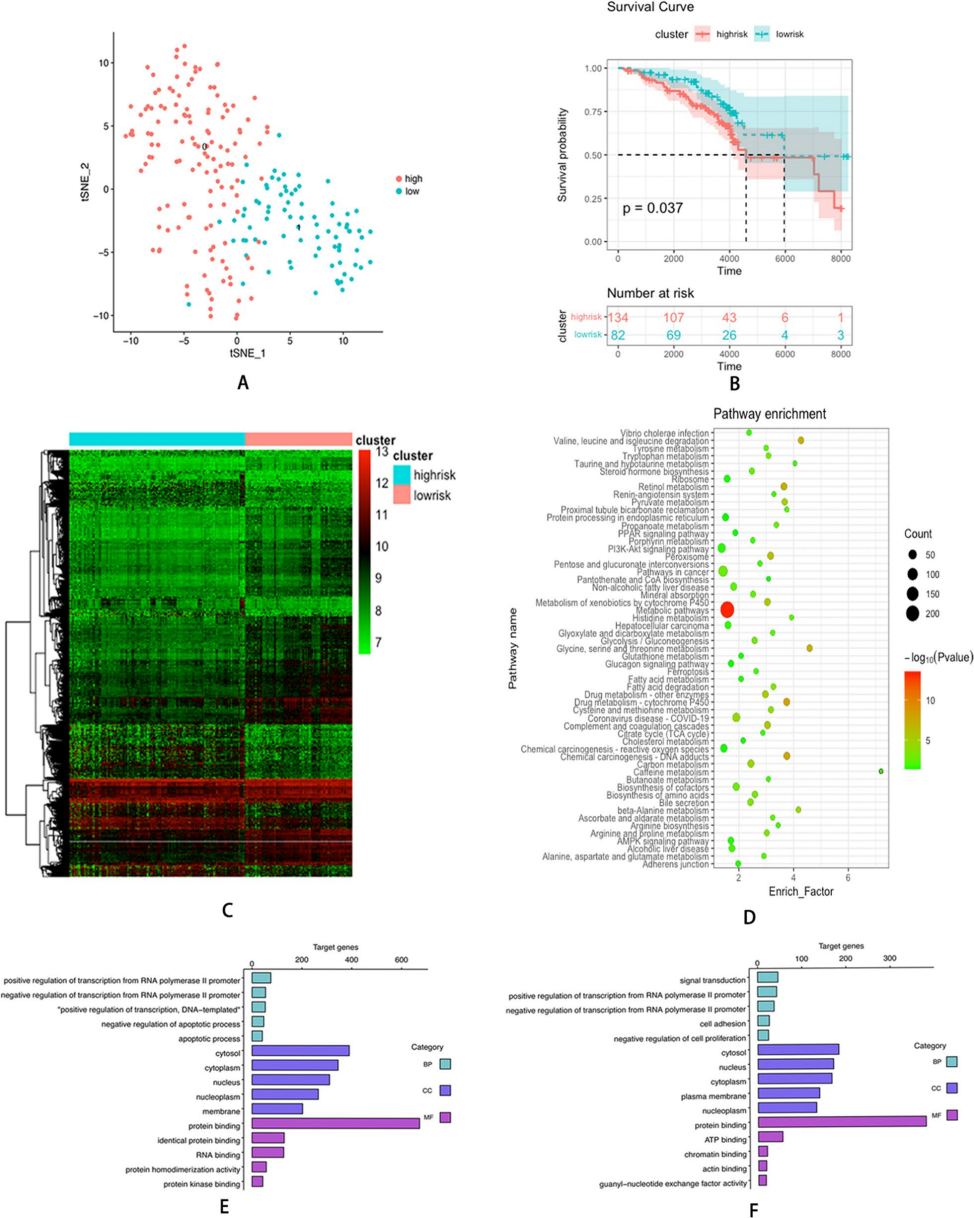
**A** UMAP clustering plot based on the expression of hypoxia genes. **B** Kaplan–Meier plot of overall survival between hypoxia^high^ cluster and hypoxia^low^ cluster. **C** The differential gene expression profiles between two clusters. **D** KEGG pathway bubble diagram of hypoxia-related DEGs. **E** GO analysis of overexpression genes of hypoxia^high^ cluster. **F** GO analysis of low expression genes of hypoxia^high^ cluster

UMAP was used to identify hypoxia^high^ and hypoxia^low^ patients (Fig. 2A). The hypoxia^high^ and hypoxia^low^ groups comprised 134 and 82 patients, respectively. The survival plot revealed that individuals with elevated hypoxia status had unfavorable prognoses (*P* = 0.037) (Fig. 2B). When comparing the expression profiles between the two clusters, 1656 DEGs were detected and considered hypoxia-related DEGs (Fig. 2C). KEGG pathway analysis indicated the enrichment of hypoxia-related DEGs in multiple pathways, including the metabolic pathway, pathway in cancer, Rap1 signaling pathway, and other pathways (Fig. 2D). Regarding biological processes (BP), overexpressed genes in the hypoxia^high^ cluster were mainly associated with the positive regulation of transcription from RNA polymerase II promoter, negative regulation of transcription from RNA polymerase II promoter, and positive regulation of transcription, DNA-templated (Fig. 2E). Regarding cellular components (CC), these genes exhibited enrichment in the cytosol, nucleus, and nucleoplasm (Fig. 2E). Molecular function (MF) analysis demonstrated that these genes were significantly associated with protein binding, identical protein binding, and RNA binding (Fig. 2E). Contrastingly, BP analysis demonstrated that low expression genes in the hypoxia^high^ cluster primarily exhibited enrichment in signal transduction, positive regulation of transcription from RNA polymerase II promoter, and negative regulation of transcription from RNA polymerase II promoter (Fig. 2F). CC analysis demonstrated that these genes exhibited enrichment in the cytosol, nucleus, and cytoplasm (Fig. 2F). MF analysis demonstrated that these genes were significantly linked to protein binding, APT binding, and chromatin binding (Fig. 2F). The clinical data of patients in the two clusters are depicted in Table 2. The findings demonstrated that high hypoxia status predicts a poorer prognosis in individuals with HCV-related early-stage cirrhosis.

**Table 2:** Basic information of HCV patients in different hypoxia-based clusters

Characteristics	Whole cohort (216)	Hypoxia ^low^ (82)	Hypoxia ^high^ (134)
Survival	150 (69.44%)	64 (78.05%)	86 (64.18%)
Survival time (days)	3373.13 ± 1525.10	3514.67 ± 1531.66	3286.52 ± 1520.29
Bilirubin ≥ 1.0 mg/dl	108 (50.00%)	38 (46.34%)	70 (52.24%)
HCC	65 (30.09%)	21 (25.61%)	44 (32.84%)

*HCC* Hepatic carcinoma, *HCV* hepatitis C virus

### High immune status indicated poor prognosis in HCV-induced early-stage fibrosis

**Fig. 3:**
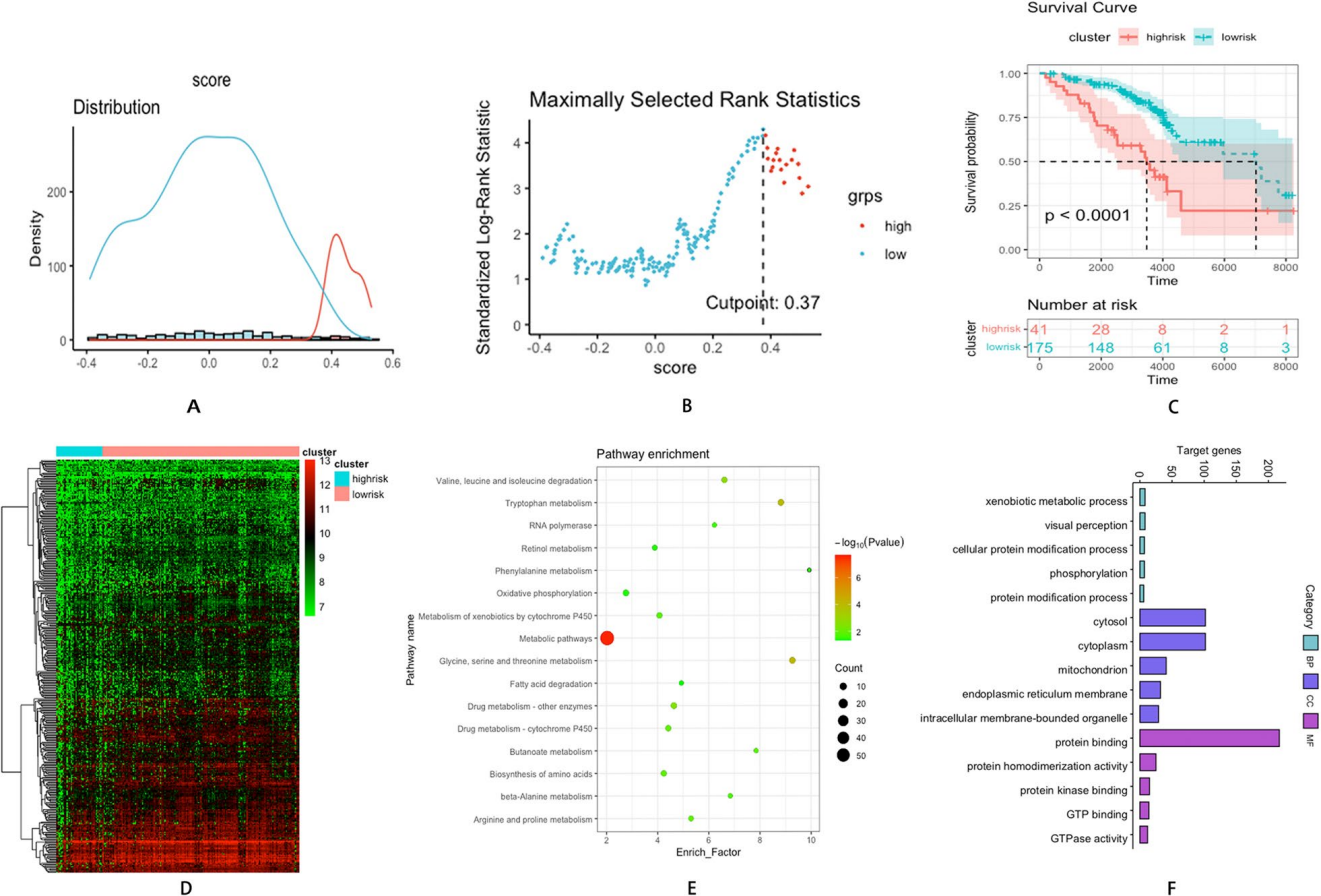
**A** Histogram based on maximally selected rank grouping. **B** The cut-off point with the maximum standard log-rank statistic was marked with a vertical dashed line. **C** Kaplan–Meier plot of overall survival between immune^high^ cluster and immune^low^ cluster. **D** The differential gene expression profiles between two groups. **E** KEGG pathway bubble diagram of immune-related DEGs **F** GO analysis of overexpression genes of immune-related DEGs

ESTIMATE was used to calculate the immune score of patients. Combining the survival time and status of patients, coefficients were calculated. The risk score was derived utilizing the formula below: 0.0022225 × Stromal Score + − 0.0002436 × Immune score. Patients were separated into immune^high^ and immune^low^ clusters using maximally selected rank statistics at a cut-off value of 0.37 (Fig. 3A, B). A total of 41 individuals were observed in the immune^high^ cluster and 175 individuals in the immune^low^ cluster. The survival plot demonstrated that individuals with high immune status indicated shorter survival time than individuals with low immune status (*P* < 0.0001) (Fig. 3C). Differential genes analysis between the two groups revealed 362 DEGs, which were considered immune-related DEGs (Fig. 3D). KEGG pathway analysis demonstrated that these genes exhibited enrichment in metabolic pathways, glycine, serine, threonine, and tryptophan metabolism (Fig. 3E). BP analysis indicated that immune-related DEGs exhibited enrichment in the xenobiotic metabolic process, visual perception, and cellular protein modification process. CC analysis highlighted that these genes were significantly linked to the cytosol, cytoplasm, and mitochondrion. MF analysis highlighted that these genes were primarily associated with protein binding, protein homodimerization activity, and protein kinase binding (Fig. 3F). The clinical data of the two groups are presented in Table 3. Collectively, the results revealed that individuals with high immune status predicted a poorer prognosis in HCV-related early-stage fibrosis.

**Table 3:** Basic information of HCV patients in different immune-based clusters

Characteristics	Whole cohort (216)	Immune ^low^ (175)	Immune ^high^ (41)
Survival	150 (69.44%)	132 (75.43%)	18 (43.90%)
Survival time (days)	3373.13 ± 1525.10	3495.29 ± 1473.06	2851.73 ± 1648.90
Bilirubin ≥ 1.0 mg/dl	108 (50.00%)	84 (48.00%)	24 (58.54%)
HCC	65 (30.09%)	48 (27.43%)	17 (41.46%)

*HCC* Hepatic carcinoma, *HCV* hepatitis C virus

### Hypoxia-immune-related DEGs in HCV-induced early-stage fibrosis

**Fig. 4:**
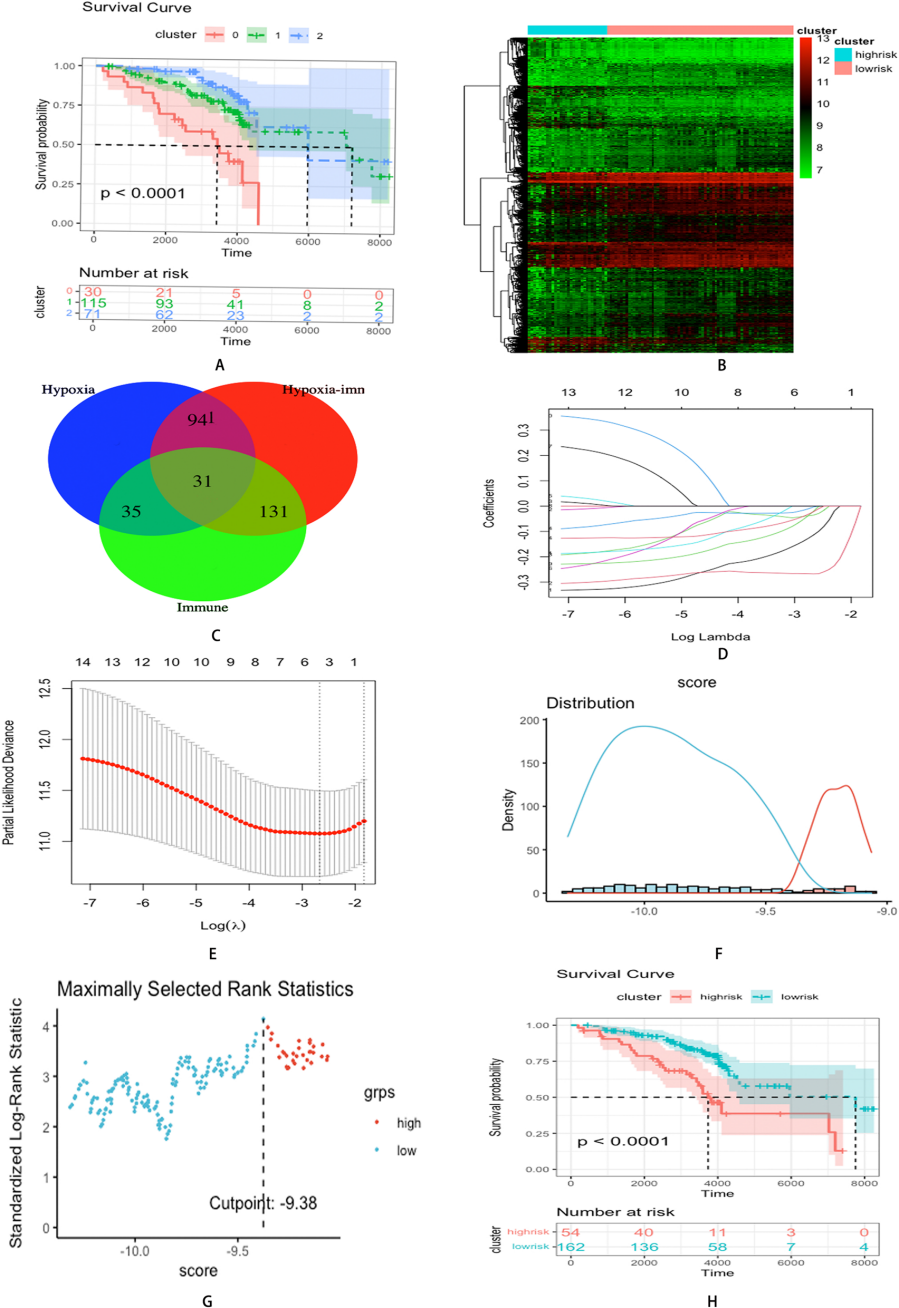
**A** Kaplan–Meier plot of overall survival among three clusters. **B** The differential gene expression profiles between hypoxia^high^immune^high^ cluster and hypoxia^low^immune^low^ cluster. **C** Venn diagrams of hypoxia-immune related DEGs. **D** LASSO coefficient profiles of 15 screened hypoxia-immune related DEGs. **E** Three-fold cross validation of lasso analysis. Error bars represented the SE. The dotted vertical lines showed the optimal values. **F** Histogram based on maximally selected rank grouping. **G** The cut-off point with the maximum standard log-rank statistic was marked with a vertical dashed line. **H** Kaplan–Meier plot of overall survival between high-risk cluster and low-risk cluster

Per the grouping results of the above analyses, individuals were further classified into three groups (hypoxia^high^immune^high^, hypoxia^low^immune^low^, and mix). Survival plot analysis demonstrated a remarkable variation in survival time among the three clusters (*P* < 0.0001) (Fig. 4A). Differential genes analysis between the hypoxia^high^immune^high^ and hypoxia^low^immune^low^ clusters (Fig. 4B) identified hypoxia-immune-related DEGs. Then, the intersection of the hypoxia-related DEGs and immune-related DEGs (Fig. 4C) revealed 31 hypoxia-immune-related DEGs. The clinical data of the individuals are depicted in Table 4.

**Table 4:** Basic information of HCV patients in different hypoxia-immune-based clusters

Characteristics	Whole cohort (216)	Hypoxia ^low^ Immune ^low^ (71)	Hypoxia ^high^ Immune ^high^ (30)
Survival	150 (69.44%)	58 (81.69%)	12 (40.00%)
Survival time (days)	3373.13 ± 1525.10	3551.83 ± 1352.96	2696.60 ± 1244.37
Bilirubin ≥ 1.0 mg/dl	108 (50.00%)	32 (45.07%)	17 (56.67%)
HCC	65 (30.09%)	18 (25.35%)	14 (46.67%)

*HCC* Hepatic carcinoma, *HCV* hepatitis C virus

### Prognosis prediction model of HCV-related early-stage cirrhosis based on hypoxia-immune-related DEGs

**Fig. 5:**
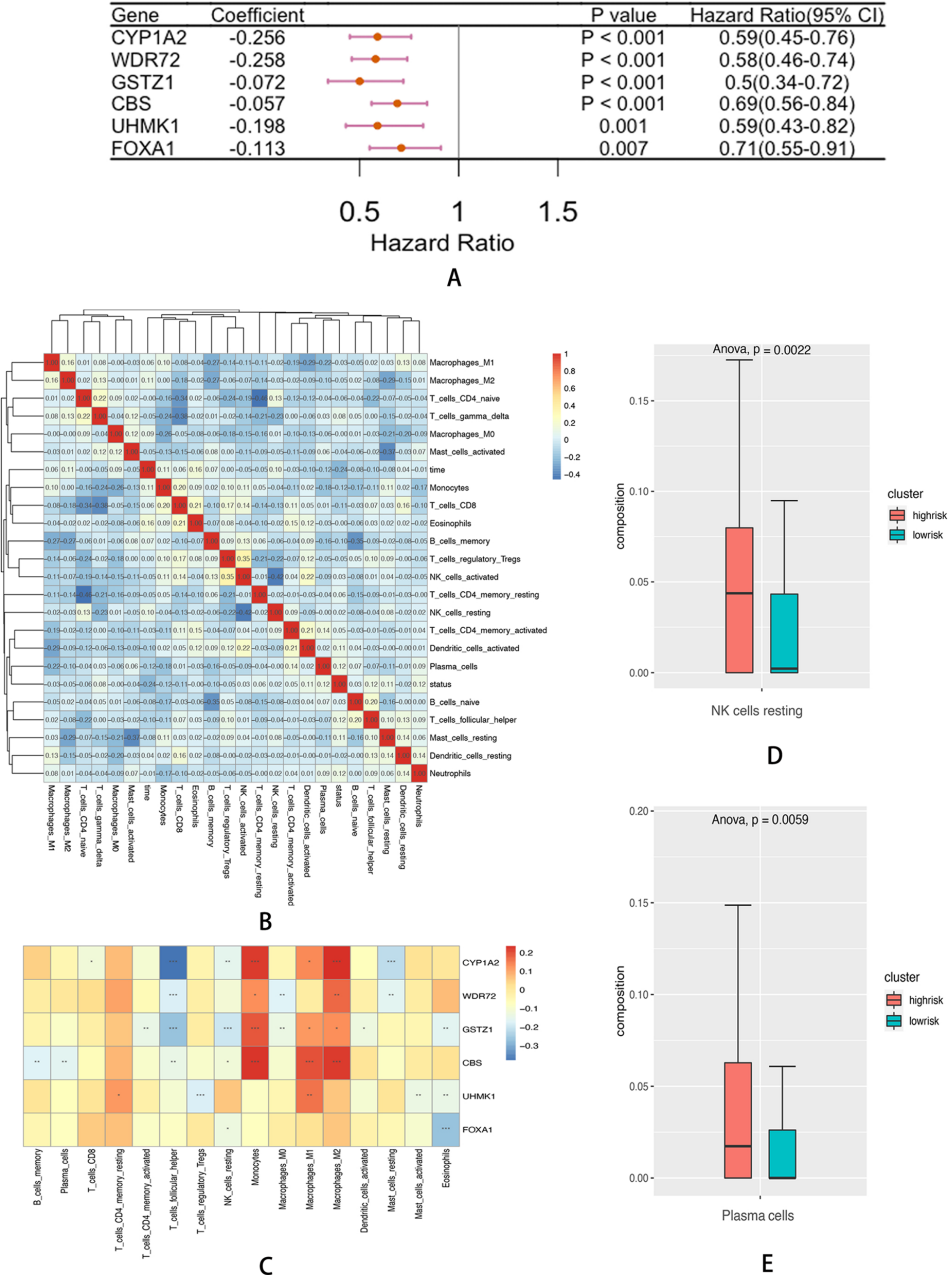
**A** Forest plot of 6 hypoxia-immune related DEGs with P < 0.01 by univariate Cox regression. **B** Heatmap showed the correlation coefficient between different immune cells. **C** Heatmap showed the correlation coefficient between immune cells and DEGs involved in Cox model (*** means P < 0.01, ** means P < 0.05, and * means P < 0.1). **D** The infiltration of NK resting cells in two clusters based on the prognostic model. **E** The infiltration of plasma cells in two clusters based on the prognostic model

Following univariate Cox analysis, 15 hypoxia-immune-related genes with *P* < 0.01 were initially identified. The LASSO method was applied to further refine the selection, resulting in the retention of six hypoxia-immune related DEGs (Fig. 4D, E). The corresponding coefficients were calculated from the multivariate Cox regression model. The risk score of each affected individual was derived using the formula: − 0.25572 × expression of CYP1A2 + (− 0.25771 × expression of WDR72) + (− 0.05747 × expression of CBS) + (− 0.0722 × expression of GSTZ1) + (− 0.19824 × expression of UHMK1 + − 0.11340 × expression of FOXA1). As per the risk score, individuals were classified into high and low-risk groups using maximally selected rank statistics, with a cut-off value of − 9.38 (Fig. 4F, G). There were 54 individuals in the high-risk cluster and 162 in the low-risk cluster. Moreover, survival analysis revealed a remarkable variation between the two clusters (*P* < 0.0001; Figs. 4H). The clinical data of the affected individuals are presented in Table 5. The forest map of the six hypoxia-immune-related DEGs is displayed in Fig. 5A.

**Table 5:** Basic information of HCV patients in hypoxia-immune-based risk models

Characteristics	Whole cohort (216)	Hypoxia ^low^ Immune ^low^ (162)	Hypoxia ^high^ Immune ^high^ (54)
Survival	150(69.44%)	123(75.93%)	27(50.00%)
Survival time (days)	3373.13 ± 1525.10	3498.77 ± 1475.46	2996.22 ± 1621.51
Bilirubin ≥ 1.0 mg/dl	108(50.00%)	79(48.77%)	29(53.70%)
HCC	65(30.09%)	45(27.78%)	20(37.04%)

*TB* Total Bilirubin, *DB* Direct Bilirubin, *IB* Indirect Bilirubin, *ALT* Alanine aminotransferase, *AST* Aspartate aminotransferase, *HCC* Hepatic carcinoma, *HCV* hepatitis C virus

### Immunocyte infiltration pattern of hypoxia-immune-related DEGs in HCV Early-stage related fibrosis

In this section, CIBERSORT analysis explored the composition of 22 immune cells. Correlation analysis revealed a general correlation between distinct immune cells (Fig. 5B). Then, the association between immune cells and hypoxia-immune-related DEGs was explored (Fig. 5C). According to the grouping of the prognosis model, a comparison of the variations in immune cells between the high- and low-risk clusters was performed. It was noted that the infiltration of resting NK cells and plasma cells was elevated in the high-risk group relative to the low-risk group (*P* = 0.0022, *P* = 0.0059, respectively; Fig. 5D, E).

### Validation of clinical samples

**Fig. 6:**
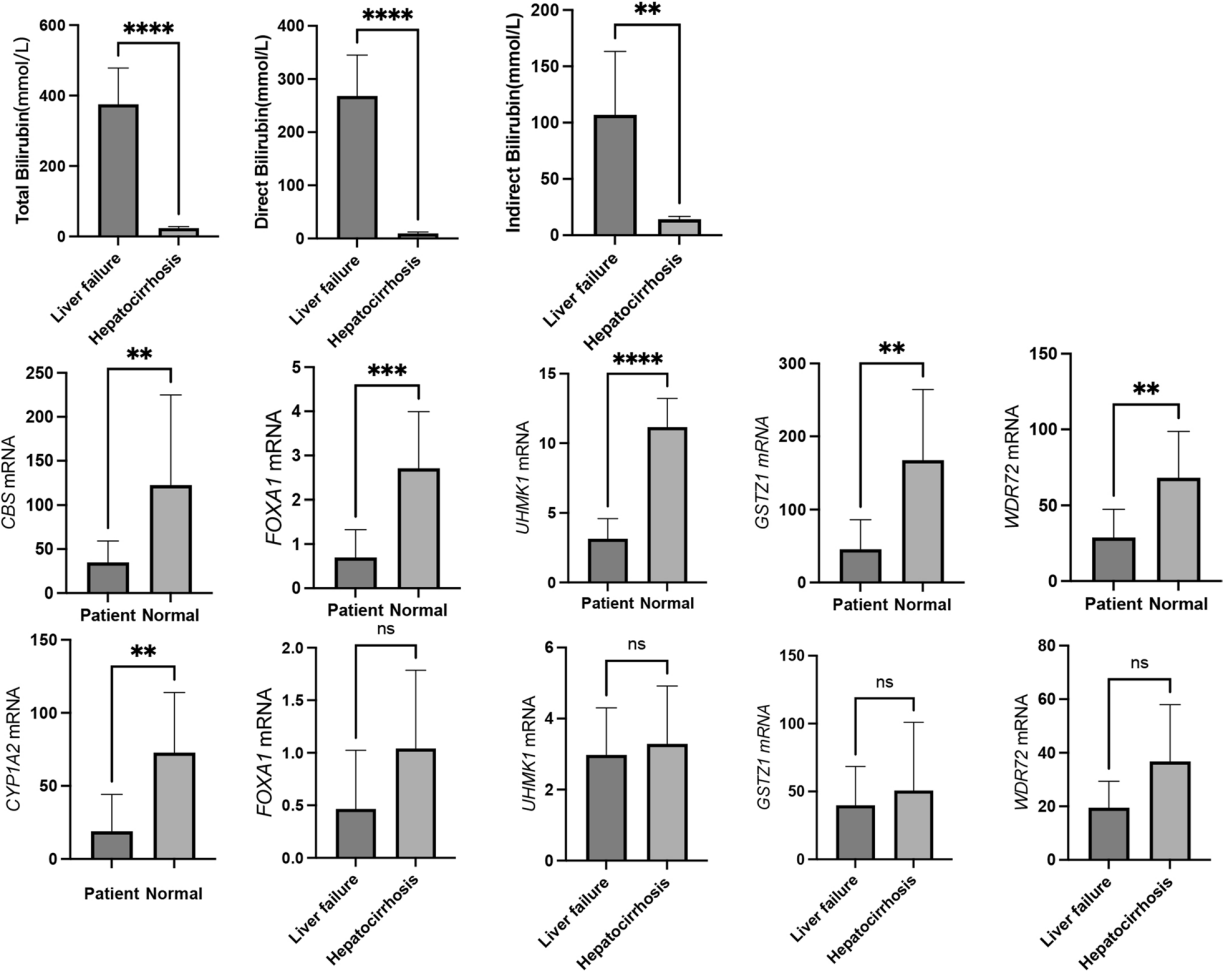
The clinical information of patients and the expression of hypoxia-immune related DEGs in clinical samples. *, **, ***, **** respectively represent P values of t-test < 0.05, < 0.01, < 0.001, < 0.0001

The expression of hypoxia-immune-related DEGs of specimens from different stages of cirrhosis were compared. The clinical data of the individuals are presented in Table 6. The levels of direct bilirubin, indirect bilirubin, and total bilirubin were higher in patients with liver failure than in patients with hepatic cirrhosis (Fig. 6). Furthermore, the liver tissues of patients with liver failure and hepatic cirrhosis exhibited downregulated expression of *CBS*, *CYP1A2*, *FOXA1*, *GSTZ1*, WDR72 and *UHMK1* when compared to healthy controls. Notably, the expression of *FOXA1, GSTZ1,* WDR72 and *UHMK1* was more upregulated in the tissues of individuals with hepatic cirrhosis than those with liver failure, although statistical significance was not achieved.

**Table 6:** The clinical information of collect samples

Characteristics	ALL (19)	Liver Failure (6)	Hepaticirrhosis (7)	Donor Liver (6)
TB	186.14 ± 194.18	375.45 ± 102.97	23.87 ± 4.50	NA
DB	129.01 ± 143.13	268.32 ± 76.87	9.60 ± 2.57	NA
IB	57.05 ± 60.32	106.97 ± 56.33	14.27 ± 2.49	NA
ALT	94.23 ± 194.61	58.50 ± 29.44	124.86 ± 269.54	NA
AST	78.31 ± 79.50	106.67 ± 55.41	54.00 ± 92.67	NA

*HCC* Hepatic carcinoma, *HCV* hepatitis C virus

### Validation of total bilirubin and hypoxia-immune-related DEGs genes in liver failure samples RNA-sequencing data

**Fig. 7:**
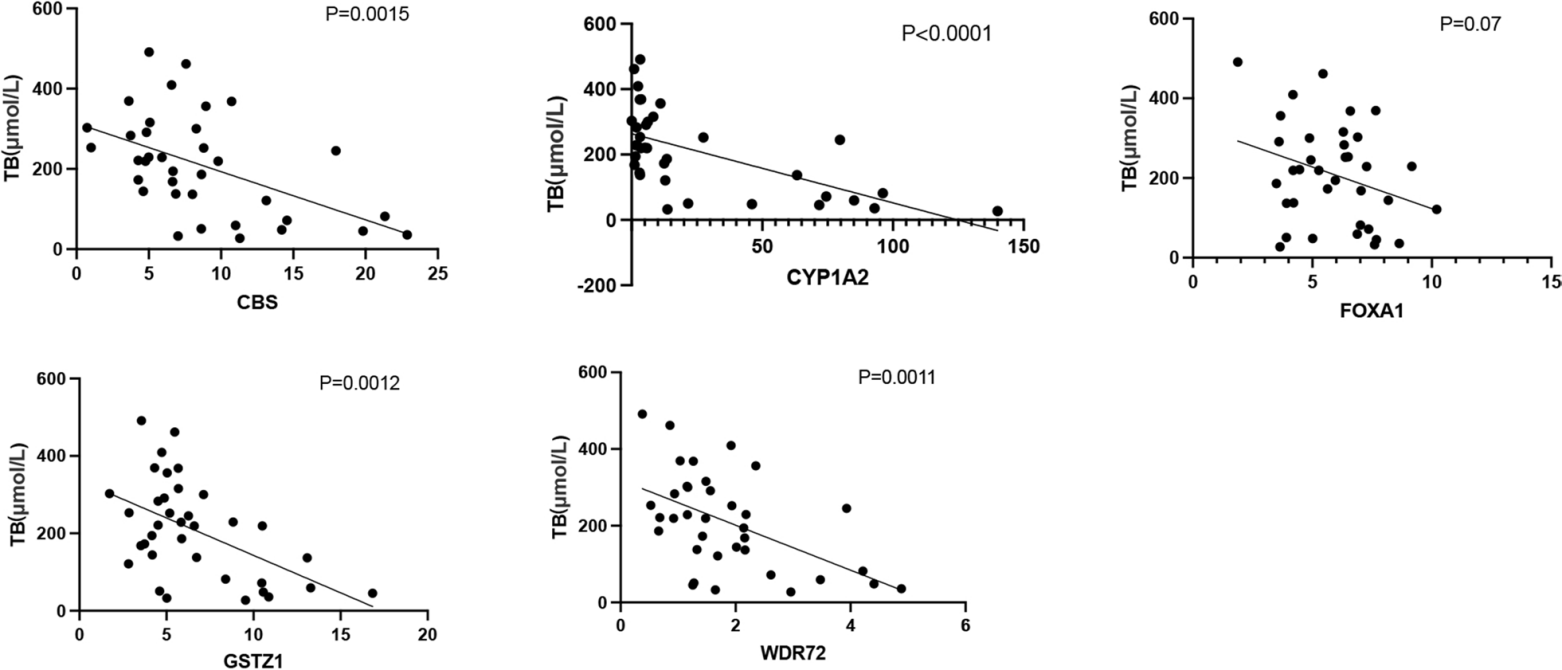
The relationship between the expression of hypoxia-immune-related DEGs and total bilirubin. *TB* total bilirubin

The relationship between the expression of hypoxia-immune-related DEGs and total bilirubin was explored. We found that the total bilirubin showed a negative correlation with the gene expression of *CBS, CYP1A2, FOXA1 and GSTZ1* (Fig. 7).

## Discussion

Currently, effective treatments for HCV-induced liver fibrosis are still lacking, highlighting the need to identify novel targets for treating patients with liver fibrosis. This research used the transcriptional profiles of the liver tissue to analyse the correlation between the prognosis of patients and the hypoxia-immune-related gene expression. It was observed that elevated hypoxia and immune status were linked to poor prognosis in individuals with HCV-induced early liver fibrosis. Furthermore, six hypoxia-immune-related genes were identified, highlighting the potential to effectively predict the prognosis of individuals with HCV-induced early fibrosis.

Previous studies have demonstrated that hypoxia and immune status are considerably linked to the advancement of liver fibrosis [6, 7]. Under hypoxic conditions, cells and tissues initiate the transcriptional induction of downstream genes to sustain physiological processes. During hypoxia, there is an increased generation of free radical oxygen, resulting in the generation of reactive oxygen species (ROS) [8]. Consequently, ROS stimulates the activation of hepatic stellate cells (HSC) and fibroblasts to aggravate the deposition of ECM and mediate chronic inflammation [9]. In individuals with HCV infection, long-term chronic immune reaction results in liver fibrosis [10, 11]. Hypoxia serves as an important microenvironment of chronic inflammation. In a normal physiological state, there exists an oxygen concentration gradient within the liver, with higher oxygen content in the portal vein area and lower oxygen content in the central vein area [12]. This oxygen gradient determines distinct functionalities of liver cells in different regions. In areas with higher oxygen content, such as the portal vein, liver cells primarily engage in ATP-consuming tasks like protein synthesis and secretion, gluconeogenesis, and beta-oxidation of fatty acids. Conversely, in regions surrounding the central vein with lower oxygen levels, liver cells undertake lower-energy tasks like detoxification, lipid synthesis, and glycoprotein synthesis. Current research has reported the relationship between oxygen gradients and liver fibrosis [13]. Yuan et al. found that an increased number of vessels in the portal tract area inhibits liver fibrosis, while an increased number of vessels around the central vein promotes liver fibrosis [14]. Fibrosis typically initiates from the portal tract or central vein, possibly associated with parenchymal liver damage and closure of liver sinusoids, leading to sustained hypoxia. The deposition of collagen at both ends alters the physiological oxygen gradient, factors that may influence the progression of fibrosis. Factors affecting tolerance to hypoxia in different areas need consideration. Meanwhile, the immune microenvironment also participates in the complex process of liver fibrosis. Consequently, further study is still needed to elucidate the specific role of oxygen gradients in liver fibrosis.

There are various types of immune cells in the development of fibrosis in the liver. Kupffer cells, which are resident macrophages, mediate liver inflammation by becoming activated and secreting cytokines in response to chronic liver injury [15]. Moreover, hypoxia stimulates the production of lipopolysaccharide (LPS) via the portal vein and systemic circulation. The combination of LPS and Toll-like receptor 4 increases the generation of chemokines, which recruits Kupffer cells and stimulates the expression of profibrogenic cytokine TGF-β by Kupffer cells [16, 17]. Thus, hypoxia not only induces the aggravation of the fibrosis but also stimulates the immune reaction that exacerbates the progression of the inflammation. Similarly, this study demonstrated that hyper immunity and hypoxia status predict poor prognosis in patients with HCV-induced early-stage liver fibrosis. CIBERSORT analysis demonstrated that the infiltration of resting NK cells and plasma cells in the hypoxia^high^immune^high^ group was greater than in the hypoxia^low^immune^low^ group. Plasma cells, also known as terminally differentiated B cells, produce antibodies in response to viral, bacterial, and parasitic infections [18]. Plasma cells are critically involved in immediate and long-lasting host immune responses [19]. Moreover, the deficiency of MYD88 signaling in B cells decreased the infiltration of dendritic cells and monocytes in CCL4-induced fibrosis mice [20]. Notably, NK cells generally display antifibrotic roles [21]. However, compared to activated NK cells, resting NK cells cannot secret IFN-γ, which is essential for inhibiting activated HSCs [22]. This study demonstrated that the high infiltration of resting NK and plasma cells is a risk factor for the aggravation of liver fibrosis.

In this research, six genes were identified, which were termed hypoxia-immune-related DEGs, to construct the prediction model. According to the validation of clinical specimens, *CBS, CYP1A2, FOXA1, GSTZ1,* WDR72 and *UHMK1* were specifically expressed in patients with liver fibrosis or hepatocirrhosis. The six genes exerted a protective effect and were more highly expressed in healthy control than patient samples. Cytochrome P450 1A2 (CYP1A2) has been widely accepted as an important metabolic enzyme drug [23]. The expression of CYP1A2 was attenuated among individuals with non-alcoholic fatty liver disease [24–29]. We found that the expression of CYP1A2 was inhibited in patients with liver fibrosis. However, the specific mechanism of CYP1A2 in liver fibrosis still requires additional investigation. Cystathionine beta-synthase (CBS) has been clearly demonstrated in lung fibrosis and renal fibrosis [30–32] The increased expression of CBS attenuated TGF-β1-induced ECM deposition in vitro*.* Under hypoxia conditions, the expression of CBS was decreased in kidney ischemia–reperfusion injury [33]. CBS could regulate the expression of HIF-1α stability [34, 35]. We found that the level of CBS had a negative correlation with the progression of fibrotic disease and hypoxia. Glutathione S-transferase zeta 1 (GSTZ1) has been clearly described in some types of cancers, such as HCC and renal cell carcinoma [36; 37; 38; 39]. Sustained Wnt/β-catenin pathway reactivation has been associated with the onset and progression of liver fibrosis [40]. We found that GSTZ1 may have a protective role in the onset and progression of liver fibrosis. Nevertheless, the specific mechanism of GSTZ1 in the progression of liver fibrosis still requires additional investigation. The forkhead box protein A 1(FOXA1) participates in the development of embryogenesis and liver organogenesis [41]. Song et al*.* revealed that FOXA1 loss led to the activation of the TGF-β pathway and epithelial-mesenchymal transition in prostate cancer [42]. Additionally, the decreased expression of FOXA1 in prostate cancer led to an increase in immunosuppressive macrophage infiltration, which is dependent on HIF-1α expression [43]. This study indicated that the elevated expression of FOXA1 is linked to a better prognosis in individuals with HCV-induced early liver fibrosis. However, the detailed mechanism stills need to be further studied. WD repeat-containing protein (WDR72) has been considered a cancer suppressor and a potential therapeutic target in renal cell carcinoma, lung cancer and amelogenesis imperfecta [44–46]. Nevertheless, the function of WDR72 in liver and organ fibrosis is yet to be elucidated. This research identified WDR72 as a hypoxia-immune-related protective DEG. Based on existing knowledge, this research represents the primary exploration of the role of WDR72 in liver fibrosis. However, its concrete function still needs to be further explored in liver fibrosis. U2AF homology motif kinase (UHMK1) has been discussed in colorectal cancer, HCC, gastric cancer, and pancreatic ductal adenocarcinoma. In these different cancer types, elevated expression levels of UHMK1 promote the proliferation of cancer cells [47–50]. However, the role of UHMK1 in liver fibrosis remains to be explored. Our research revealed that the elevated expression level of UHMK1 in patients with HCV-induced early liver fibrosis predicted a great prognosis. Nevertheless, the role of UHMK1 in hypoxia and liver fibrosis needs to be discussed further. The mechanism of CBS, CYP1A2, FOXA1, GSTZ1, WDR72 and UHMK1 in the progression of liver fibrosis is still unclear. Further exploration of the underlying mechanisms will help to find potential targets for the treatment of liver cirrhosis.

The current research identified six hypoxia-immune-related genes in patients with HCV-induced early-stage liver fibrosis. Our model effectively predicts the prognosis of patients. The validation of clinical samples strongly supports the results. However, this research still has some limitations. Firstly, the clinical samples were limited, with only six samples in each group. More specimens are required to verify the study results in the future. Secondly, the infiltration of immune cells was not validated using flow cytometry. Hence, future studies should detect the infiltration of NK cells and B cells. Thirdly, although the research detected a series of hypoxia-immune-related DEGs, basic research to explore the mechanisms of each gene was not conducted.

## Conclusion

Elevated hypoxia and immune status indicated a poorer prognosis for individuals with HCV-induced early-stage liver fibrosis. Based on the six hypoxia-immune-related genes, the prognostic model could effectively predict the prognosis and progression of liver fibrosis. Notably, this study provides novel directions for the future diagnosis and treatment of liver fibrosis.

## Data Availability

The datasets used and/ or analysed during the current study are available from the corresponding author on reasonable request.

## References

[1] Sebastiani G, Gkouvatsos K, Pantopoulos K (2014). Chronic hepatitis C and liver fibrosis. World J Gastroenterol.

[2] Roehlen N, Crouchet E, Baumert TF (2020). Liver fibrosis: mechanistic concepts and therapeutic perspectives. Cells.

[3] Ginès P, Krag A, Abraldes JG, Solà E, Fabrellas N, Kamath PS (2021). Liver cirrhosis. Lancet.

[4] Kietzmann T, Dimova EY, Flügel D, Scharf JG (2006). Oxygen: modulator of physiological and pathophysiological processes in the liver. Z Gastroenterol.

[5] Parola M, Pinzani M (2019). Liver fibrosis: Pathophysiology, pathogenetic targets and clinical issues. Mol Aspects Med.

[6] Foglia B, Novo E, Protopapa F, Maggiora M, Bocca C, Cannito S, Parola M (2021). Hypoxia, hypoxia-inducible factors and liver fibrosis. Cells.

[7] Cai J, Hu M, Chen Z, Ling Z (2021). The roles and mechanisms of hypoxia in liver fibrosis. J Transl Med.

[8] Borle AB, Barsic M (1995). Chemical hypoxia increases cytosolic Ca2+ and oxygen free radical formation. Cell Calcium.

[9] Luangmonkong T, Suriguga S, Mutsaers HAM, Groothuis GMM, Olinga P, Boersema M (2018). Targeting oxidative stress for the treatment of liver fibrosis. Rev Physiol Biochem Pharmacol.

[10] Stuart JD, Salinas E, Grakoui A (2021). Immune system control of hepatitis C virus infection. Curr Opin Virol.

[11] Troeger JS, Schwabe RF (2011). Hypoxia and hypoxia-inducible factor 1alpha: potential links between angiogenesis and fibrogenesis in hepatic stellate cells. Liver Int.

[12] Kietzmann T (2017). Metabolic zonation of the liver: The oxygen gradient revisited. Redox Biol.

[13] Kietzmann T (2019). Liver zonation in health and disease: hypoxia and hypoxia-inducible transcription factors as concert masters. Int J Mol Sci.

[14] Lin Y, Dong MQ, Liu ZM, Xu M, Huang ZH, Liu HJ, Gao Y, Zhou WJ (2022). A strategy of vascular-targeted therapy for liver fibrosis. Hepatology.

[15] Koyama Y, Brenner DA (2017). Liver inflammation and fibrosis. J Clin Invest.

[16] Schwabe RF, Seki E, Brenner DA (2006). Toll-like receptor signaling in the liver. Gastroenterology.

[17] Brun P, Castagliuolo I, Pinzani M, Palù G, Martines D (2005). Exposure to bacterial cell wall products triggers an inflammatory phenotype in hepatic stellate cells. Am J Physiol Gastrointest Liver Physiol.

[18] Ripperger TJ, Bhattacharya D (2021). Transcriptional and metabolic control of memory B Cells and plasma cells. Annu Rev Immunol.

[19] Novobrantseva TI, Majeau GR, Amatucci A, Kogan S, Brenner I, Casola S, Shlomchik MJ, Koteliansky V, Hochman PS, Ibraghimov A (2005). Attenuated liver fibrosis in the absence of B cells. J Clin Invest.

[20] Thapa M, Chinnadurai R, Velazquez VM, Tedesco D, Elrod E, Han JH, Sharma P, Ibegbu C, Gewirtz A, Anania F, Pulendran B, Suthar MS, Grakoui A (2015). Liver fibrosis occurs through dysregulation of MyD88-dependent innate B-cell activity. Hepatology.

[21] Wolter F, Glassner A, Kramer B, Kokordelis P, Finnemann C, Kaczmarek DJ, Goeser F, Lutz P, Nischalke HD, Strassburg CP, Spengler U, Nattermann J (2015). Hypoxia impairs anti-viral activity of natural killer (NK) cells but has little effect on anti-fibrotic NK cell functions in hepatitis C virus infection. J Hepatol.

[22] Tsuchida T, Friedman SL (2017). Mechanisms of hepatic stellate cell activation. Nat Rev Gastroenterol Hepatol.

[23] Wójcikowski J, Daniel WA (2009). Perazine at therapeutic drug concentrations inhibits human cytochrome P450 isoenzyme 1A2 (CYP1A2) and caffeine metabolism–an in vitro study. Pharmacol Rep.

[24] Zhang XB, Chen XY, Chiu KY, He XZ, Wang JM, Zeng HQ, Zeng Y (2022). Intermittent hypoxia inhibits hepatic CYP1a2 expression and delays aminophylline metabolism. Evid Based Complement Alternat Med.

[25] Zhang XB, Zeng YM, Chen XY, Zhang YX, Ding JZ, Xue C (2018). Decreased expression of hepatic cytochrome P450 1A2 (CYP1A2) in a chronic intermittent hypoxia mouse model. J Thorac Dis.

[26] Wuensch T, Heucke N, Wizenty J, Quint J, Sinn B, Arsenic R, Jara M, Kaffarnik M, Pratschke J, Stockmann M (2019). Hepatic CYP1A2 activity in liver tumors and the implications for preoperative volume-function analysis. Am J Physiol Gastrointest Liver Physiol.

[27] Fisher CD, Lickteig AJ, Augustine LM, Ranger-Moore J, Jackson JP, Ferguson SS, Cherrington NJ (2009). Hepatic cytochrome P450 enzyme alterations in humans with progressive stages of nonalcoholic fatty liver disease. Drug Metab Dispos.

[28] Shi LX, Wang X, Wu Q, Sun X, Wan Z, Li L, Li K, Li X, Li Y, Zhang QY, Wu JP, Chen HY (2017). Hepatic Cyp1a2 expression reduction during inflammation elicited in a rat model of intermittent hypoxia. Chin Med J (Engl).

[29] Fradette C, Bleau AM, Pichette V, Chauret N, Du Souich P (2002). Hypoxia-induced down-regulation of CYP1A1/1A2 and up-regulation of CYP3A6 involves serum mediators. Br J Pharmacol.

[30] Hamelet J, Maurin N, Fulchiron R, Delabar JM, Janel N (2007). Mice lacking cystathionine beta synthase have lung fibrosis and air space enlargement. Exp Mol Pathol.

[31] Yuan X, Zhang J, Xie F, Tan W, Wang S, Huang L, Tao L, Xing Q, Yuan Q (2017). Loss of the protein cystathionine beta-synthase during kidney injury promotes renal tubulointerstitial fibrosis. Kidney Blood Press Res.

[32] Jung KJ, Jang HS, Kim JI, Han SJ, Park JW, Park KM (1832). Involvement of hydrogen sulfide and homocysteine transsulfuration pathway in the progression of kidney fibrosis after ureteral obstruction. Biochim Biophys Acta.

[33] Wang P, Isaak CK, Siow YL (2014). Downregulation of cystathionine beta-synthase and cystathionine gamma-lyase expression stimulates inflammation in kidney ischemia-reperfusion injury. Physiol Rep.

[34] Takano N, Peng YJ, Kumar GK, Luo W, Hu H, Shimoda LA, Suematsu M, Prabhakar NR, Semenza GL (2014). Hypoxia-inducible factors regulate human and rat cystathionine beta-synthase gene expression. Biochem J.

[35] Dey A, Prabhudesai S, Zhang Y, Rao G, Thirugnanam K, Hossen MN, Dwivedi SKD, Ramchandran R, Mukherjee P, Bhattacharya R (2020). Cystathione β-synthase regulates HIF-1α stability through persulfidation of PHD2. Sci Adv.

[36] Fernández-Cañón JM, Peñalva MA (1998). Characterization of a fungal maleylacetoacetate isomerase gene and identification of its human homologue. J Biol Chem.

[37] Li J, Wang Q, Yang Y, Lei C, Yang F, Liang L, Chen C, Xia J, Wang K, Tang N (2019). GSTZ1 deficiency promotes hepatocellular carcinoma proliferation via activation of the KEAP1/NRF2 pathway. J Exp Clin Cancer Res.

[38] Wang Q, Bin C, Xue Q, Gao Q, Huang A, Wang K, Tang N (2021). GSTZ1 sensitizes hepatocellular carcinoma cells to sorafenib-induced ferroptosis via inhibition of NRF2/GPX4 axis. Cell Death Dis.

[39] Wang J, Chang H, Su M, Qiao Y, Sun H, Zhao Y, Zhang S, Shan C (2022). Identification of HGD and GSTZ1 as biomarkers involved metabolic reprogramming in kidney renal clear cell carcinoma. Int J Mol Sci.

[40] Guo Y, Xiao L, Sun L, Liu F (2012). Wnt/beta-catenin signaling: a promising new target for fibrosis diseases. Physiol Res.

[41] Friedman JR, Kaestner KH (2006). The Foxa family of transcription factors in development and metabolism. Cell Mol Life Sci.

[42] Song B, Park SH, Zhao JC, Fong KW, Li S, Lee Y, Yang YA, Sridhar S, Lu X, Abdulkadir SA, Vessella RL, Morrissey C, Kuzel TM, Catalona W, Yang X, Yu J (2019). Targeting FOXA1-mediated repression of TGF-beta signaling suppresses castration-resistant prostate cancer progression. J Clin Invest.

[43] Wang X, Brea L, Lu X, Gritsina G, Park SH, Xie W, Zhao JC, Yu J (2022). FOXA1 inhibits hypoxia programs through transcriptional repression of HIF1A. Oncogene.

[44] Zou Y, Lu Q, Yao Q, Dong D, Chen B (2020). Identification of novel prognostic biomarkers in renal cell carcinoma. Aging.

[45] Lee SK, Seymen F, Lee KE, Kang HY, Yildirim M, Tuna EB, Gencay K, Hwang YH, Nam KH, De La Garza RJ, Hu JC, Simmer JP, Kim JW (2010). Novel WDR72 mutation and cytoplasmic localization. J Dent Res.

[46] Ouyang X, Shi X, Huang N, Yang Y, Zhao W, Guo W, Huang Y (2022). WDR72 enhances the stemness of lung cancer cells by activating the AKT/HIF-1alpha signaling pathway. J Oncol.

[47] Luo Y, Han S, Yan B, Ji H, Zhao L, Gladkich J, Herr I (2022). UHMK1 is a novel marker for personalized prediction of pancreatic cancer prognosis. Front Oncol.

[48] Gao X, Bao W, Bai J, Fan K, Li L, Li Y (2022). UHMK1 aids colorectal cancer cell proliferation and chemoresistance through augmenting IL-6/STAT3 signaling. Cell Death Dis.

[49] Feng X, Ma D, Zhao J, Song Y, Zhu Y, Zhou Q, Ma F, Liu X, Zhong M, Liu Y, Xiong Y, Qiu X, Zhang Z, Zhang H, Zhao Y, Zhang K, Hong X, Zhang Z (2020). UHMK1 promotes gastric cancer progression through reprogramming nucleotide metabolism. EMBO J.

[50] Wei T, Weiler SME, Toth M, Sticht C, Lutz T, Thomann S, De La Torre C, Straub B, Merker S, Ruppert T, Marquardt J, Singer S, Gretz N, Schirmacher P, Breuhahn K (2019). YAP-dependent induction of UHMK1 supports nuclear enrichment of the oncogene MYBL2 and proliferation in liver cancer cells. Oncogene.

